# Growth factor, energy and nutrient sensing signalling pathways in metabolic ageing

**DOI:** 10.1007/s10522-017-9724-6

**Published:** 2017-08-09

**Authors:** Lucia Bettedi, Lazaros C. Foukas

**Affiliations:** 1 Institute of Healthy Ageing and Department of Genetics, Evolution and Environment, University College London, London, UK; 2 Present Address: Cell Biology and Neurobiology Branch, National Institutes of Child Health and Human Development, National Institutes of Health, Bethesda, MD USA

**Keywords:** Ageing, PI3K, Insulin resistance, Metabolism, Obesity, Diabetes

## Abstract

The field of the biology of ageing has received increasing attention from a biomedical point of view over the past decades. The main reason has been the realisation that increases in human population life expectancy are accompanied by late onset diseases. Indeed, ageing is the most important risk factor for a number of neoplastic, neurodegenerative and metabolic pathologies. Advances in the knowledge of the genetics of ageing, mainly through research in model organisms, have implicated various cellular processes and the respective signalling pathways that regulate them in cellular and organismal ageing. Associated with ageing is a dysregulation of metabolic homeostasis usually manifested as age-related obesity, diminished insulin sensitivity and impaired glucose and lipid homeostasis. Metabolic deterioration contributes to the ageing phenotype and metabolic pathologies are thought to be one of the main factors limiting the potential for lifespan extension. Great efforts have been directed towards identifying pharmacological interventions with the potential to improve healthspan and a number of natural and synthetic compounds have shown promise in achieving beneficial metabolic effects.

## Introduction

Cell signalling pathways process cues from the extracellular environment and signals of cellular status to ensure cells respond appropriately to maintain their homeostasis. Metabolic homeostasis is a key component of cellular and organismal homeostasis. In multicellular organisms, metabolic homeostasis requires the coordinate response of distinct cell and tissue types. Cell signalling pathways that sense the availability of nutrients and the energy status of the cells communicate with hormonal and growth factor signalling pathways to co-ordinately regulate whole body metabolic homeostasis. Ageing results in gradual deterioration of various cellular functions including of metabolic regulation. The age-related decline in metabolic homeostasis is likely an important contributing factor to general organismal ageing, as a number of interventions, genetic and pharmacological, affecting the activity of metabolic pathways also affect the rate of ageing. Indeed, the Insulin/IGF-1 Signalling (IIS) pathway and the mechanistic Target Of Rapamycin (mTOR) pathway are the most extensively studied pathways shown to regulate lifespan and healthspan in a number of model organisms (Fontana et al. 2010; Kenyon 2010). Moreover, calorie restriction, the most potent environmental intervention shown to extend lifespan and healthspan in a number of species, is accompanied by alterations in the insulin/IGF-1 circulating levels, whereas inhibition of the mTOR pathway has been shown to have both common and distinct effects with calorie restriction (Kaeberlein and Kennedy 2009; Miller et al. 2014).

The relationship between ageing and metabolic regulation is bidirectional: Ageing impairs the activity of key metabolic signalling pathways and the ensuing metabolic dysregulation results in accelerated ageing. For example, age-related impairment in the activity of the insulin signalling pathway results in insulin resistance. The ensuing hyperglycemia, as a result of dysregulated glucose clearance, promotes formation of advanced glycation end products (AGEs), which in turn cause tissue damage further exacerbating metabolic dysregulation and accelerating the organismal ageing process (Semba et al. 2010).

The finding that loss-of-function mutations in genes encoding components of the IIS pathway can improve health span and in many cases the metabolic profile of aged mice is seemingly paradoxical (Barzilai et al. 2012). Various potential mechanisms have been proposed to explain this phenomenon. An important implication of these findings is that pharmacological targeting of metabolic signalling pathways can produce beneficial metabolic effects and ameliorate age-related metabolic pathologies. The activity of cell signalling pathways can readily be manipulated, as a large number of their component molecules are enzymes such as kinases and phosphatases, which can be inhibited or activated with the use of natural or synthetic compounds.

Here we summarise some key findings on the role of signalling pathways in metabolic homeostasis over the course of ageing, generated mainly through research in model organisms, and the evidence supporting pharmacological manipulation of these pathways as a means to improve metabolic health at old age.

## Calorie restriction in lifespan and healthspan

**Fig. 1:**
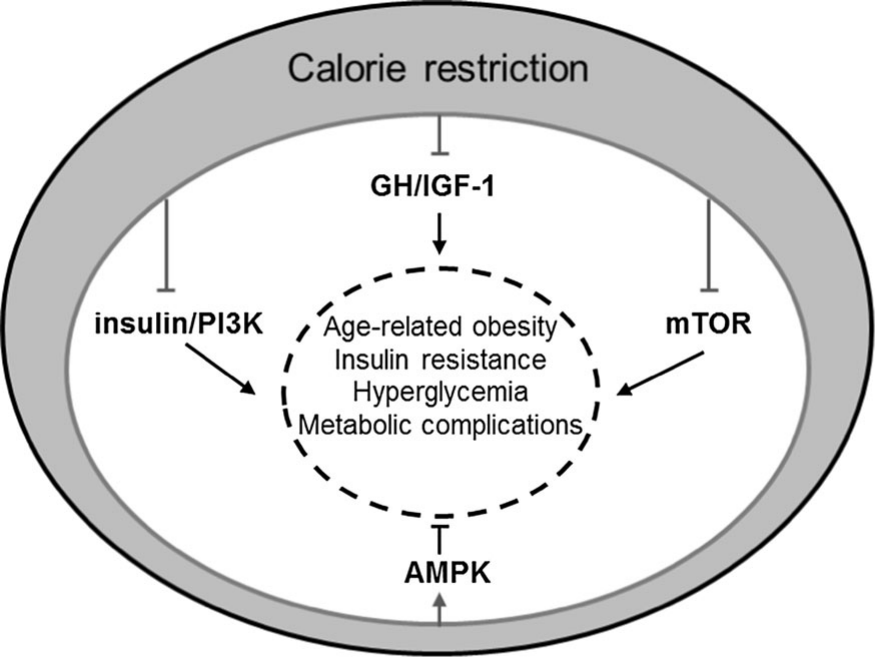
Signalling pathways implicated in age-related metabolic decline. Calorie restriction is the most potent environmental intervention that improves the metabolic profile and extends healthspan and lifespan of various animal species. Calorie restriction is thought to be suppressing the GH/IGF-1, insulin/PI3K, and mTOR pathways and activating the AMPK pathway. These pathways provide potential targets for therapeutic intervention to improve metabolic homeostasis at old age. Pointed *arrows* indicate activatory and blunt *arrows* inhibitory actions

Calorie restriction is the most potent environmental intervention known to increase lifespan and healthspan in a number of species including primates (Colman et al. 2014). The molecular mechanisms underlying the beneficial effects of calorie restriction on lifespan and healthspan have been extensively studied and debated over the years (Masoro 2009). Various mechanisms have been proposed to explain the effects of calorie restriction ranging from enhanced stress resistance and improved proteostasis (Mitchell et al. 2016) to reduced inflammation (Chung et al. 2002). With regards to metabolic effects, calorie restricted animals are normally leaner and more insulin sensitive and glucose tolerant than ad libitum fed animals. It has recently been reported that calorie restriction induces browning of the adipose tissue with profound beneficial metabolic consequences (Fabbiano et al. 2016). Also calorie restriction was recently reported to protect from accelerated ageing induced by DNA-repair deficiency (Vermeij et al. 2016). Significant progress has been made in the identification of the signalling pathways mediating the effects of calorie restriction (Fig. 1). Growth factor, energy and nutrient sensing pathways likely have a prominent role in mediating the effects of calorie restriction (Anderson and Weindruch 2010; Lopez-Lluch and Navas 2016). The fact that circulating IGF-1 and insulin levels in calorie restricted animals are lower than those in ad libitum fed ones (Argentino et al. 2005; Huffman et al. 2008; Weiss et al. 2006) has pointed to a potential role for the somatotropic (Growth Hormone (GH)/IGF-1) axis and the IIS pathways in the mediation of the effects of calorie restriction. Genetic evidence has lent further support for a role of somatotropic signalling (Bonkowski et al. 2006). Also, the mTOR and AMP-activated protein kinase (AMPK) pathways together with sirtuins, a family of NAD-dependent deacetylases have also been implicated as effectors of the benefits associated with calorie restriction. In line with genetic studies, unbiased gene expression analyses in tissues of calorie restricted animals have revealed that genes encoding components of the somatotropic and the IIS pathways as well as genes involved in metabolic processes and energy metabolism are consistently part of the molecular signature of calorie restriction (Plank et al. 2012). Therefore, pharmacological agents targeting components of these pathways might have the potential to mimic the beneficial effects of calorie restriction. The field of calorie restriction mimetic compounds has been an area of intensive research, as it holds great promise for therapeutic applications in the combat against age-related diseases. Below, we briefly summarise the key evidence implicating these cell signalling pathways in lifespan and healthspan extension and we discuss the progress in pharmacological targeting of these pathways with a focus on improvements in metabolic homeostasis.

## Growth factor, energy and nutrient sensing pathways in metabolic regulation and ageing

**Fig. 2:**
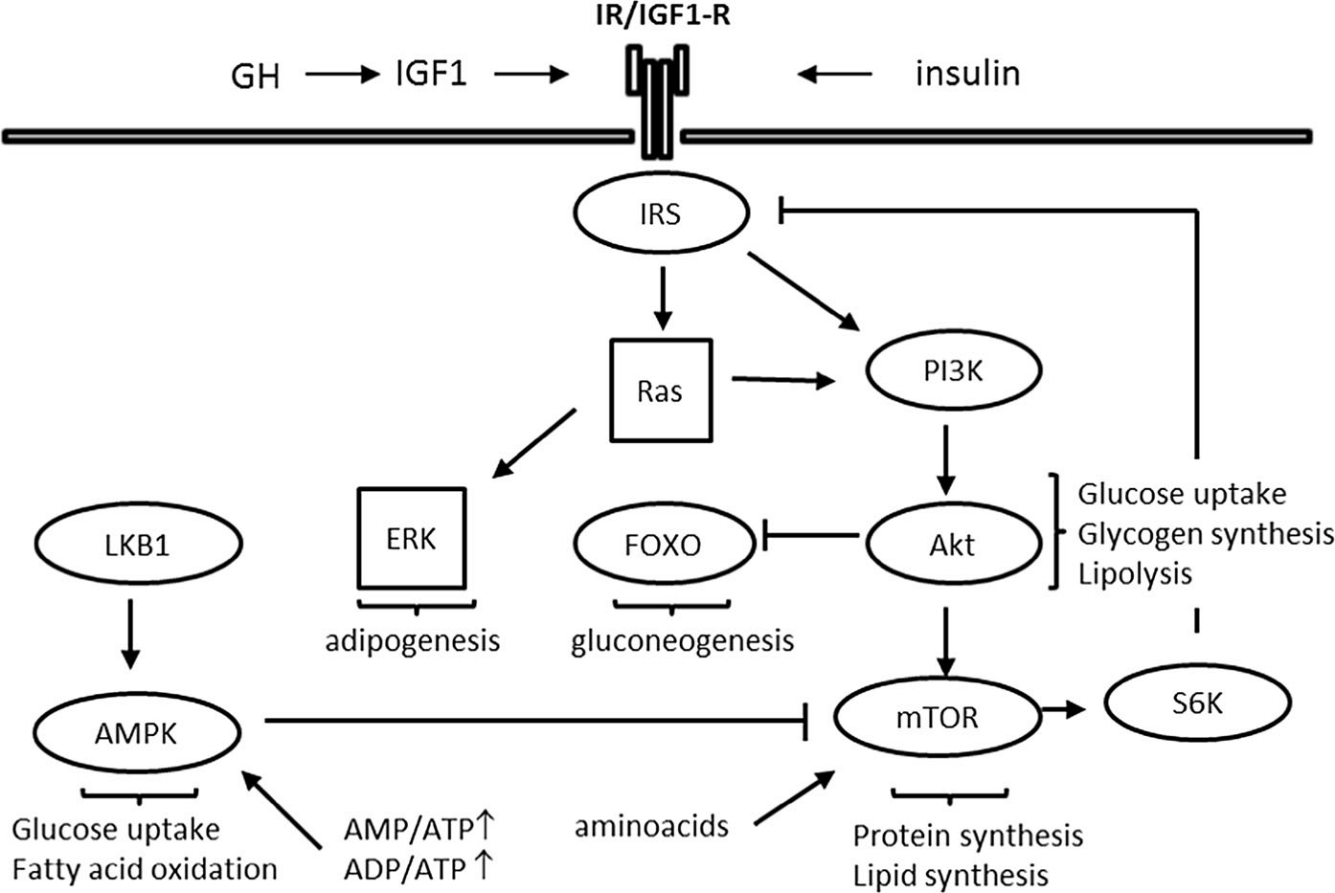
Interrelationships between growth factor, nutrient availability and energy sensing pathways in metabolic regulation in health. The Ras/ERK and PI3K/Akt pathways are activated upon insulin/IGF1 stimulation. Akt, a key effector of insulin/PI3K signalling mediates most of the metabolic actions of insulin, notably stimulation of glucose uptake and glycogen synthesis and inhibition of lipolysis. The mTOR pathway integrates signals from growth factor stimulation (via Akt), aminoacid availability and energy status (via AMPK). mTOR-activated S6K1 is a key component of a feedback loop that downregulates insulin's signal. FOXO transcription factors, which upon phosphorylation by Akt are inhibited through nuclear exclusion, also have metabolic roles, prominently in the regulation of gluconeogenesis. AMPK is activated by low energy (high AMP/ATP and/or ADP/ATP ratio) stress via phosphorylation by LKB1 and promotes glucose uptake and fatty acid oxidation. The majority of the molecular components of these pathways have been omitted from the schematic for simplicity

The main cell signalling pathways that have been implicated in the modulation of the rate of ageing have at the same time important roles in metabolic regulation. These are the somatotropic axis, insulin/IGF-1, mTOR and AMPK signalling pathways. These pathways are interlinked to ensure coordinate regulation and fine-tuning of cellular metabolic responses in line with cellular energy status, nutrient availability and hormonal/growth factor signalling input (Fig. 2). Feedback loops operate within the pathways to regulate signal intensity and duration. A key feedback mechanism for downregulation of IIS involves phosphorylation of the Insulin Receptor Substrates (IRS) by p70 ribosomal protein S6-kinase-1 (S6K1) following activation of mTOR (Harrington et al. 2004; Shah et al. 2004). In fact, sustained activation of S6K1 and various other stress-induced serine/threonine kinases is thought to be a major cellular mechanism in the development of insulin resistance (Tanti and Jager 2009). Key mediators of the metabolic effects of the IIS pathway are Phosphoinositide 3-Kinase (PI3K) and its downstream effectors, serine/threonine kinase Akt and FOXO transcription factors (Whiteman et al. 2002). FOXO transcription factors are essential mediators of the lifespan extending effects of IIS attenuation (Martins et al. 2016). Consistent with this, the gene encoding FOXO3 is one of few human genes consistently associated with longevity in a number of distinct populations (Morris et al. 2015). FOXO transcription factors have multiple metabolic effects. Notably, FOXO1 plays an essential role in the regulation of hepatic glucose production (Gross et al. 2008). mTOR’s most extensively studied role is in the regulation of protein translation. mTOR key effectors in this process are the above mentioned S6K1 and the translational repressor eIF4E-Binding Protein 1 (4EBP1). mTOR has also prominent roles in lipid biosynthesis (Caron et al. 2015).

Closely intertwined with the IIS and mTOR pathways is the Liver Kinase B (LKB) 1/AMPK pathway, which plays a role in energy status sensing (Garcia and Shaw 2017). Overexpression of one of the AMPK subunits has been shown to increase lifespan in *C. elegans* (Apfeld et al. 2004). AMPK activation brings about beneficial metabolic effects mainly by promoting glucose uptake and fatty acid oxidation. AMPK activation is thought to mediate the effects of the anti-diabetic biguanide drug metformin at least in part, as additional mechanisms have been shown to underlie metformin's effects in the liver; notably suppression of gluconeogenesis through inhibition of mitochondrial glycerophosphate dehydrogenase (Madiraju et al. 2014) and antagonism of glucagon action through accumulation of AMP and consequent inhibition of adenylyl cyclase (Miller et al. 2013).

The Ras/Extracellular Signal Regulated Kinase (ERK) pathway is an essential pathway in transmission of mitogenic signalling, which is also activated downstream the insulin/IGF-1 receptor via IRS. The Ras/ERK pathway has also been implicated in the modulation of lifespan (Slack 2017). It has recently been reported that administration of trametinib, an inhibitor of ERK activation, extends the lifespan of *D. melanogaster* (Slack et al. 2015). A key downstream effector of the pathway in lifespan extension is AOP, a transcriptional repressor of the ETS family. Whether a similar lifespan extending effect of ERK inhibition could be attainable in mammalian species remains to be seen. Evidence for metabolic effects of Ras/ERK pathway perturbations, at least under obesogenic conditions, has been presented before and it is summarised in (Slack 2017). However, the potential metabolic effects of long-term administration of this inhibitor in mammalian species also remain to be seen.

The somatotropic, insulin/IGF-1 and mTOR signalling pathways have been extensively studied in the context of ageing and age-related metabolic homeostasis over the years and they are discussed below in more detail, together with the role of sirtuins, a class of histone deacetylases, which have emerged as important regulators of cellular energy homeostasis.

### Somatotropic signalling in ageing and metabolism

Signalling via the growth hormone/insulin-like growth factor-1 (GH/IGF-1) axis, known as the somatotropic axis, is essential for body growth. IGF-1 is produced by the liver upon stimulation by GH released from the anterior pituitary gland. Loss-of-function mutations in components of the somatotropic axis have been shown to affect longevity in mammals (Junnila et al. 2013). A number of mouse mutants with spontaneous or targeted loss-of-function mutations that diminish production or impair sensing of GH have been described (Brown-Borg 2015). All these mutants have smaller body size and substantially extended lifespan. The first group of this type of mutants to be studied were hypopituitary mice, such as Snell and Ames dwarf mice which bear the *Pit*-*1* ^*dw*^ and the *Prop*-*1* ^*df*^ (Prophet of Pit-1) gene mutations, respectively. Both mutations affect the activity of the Pit-1 transcription factor, which is required for proper development of the anterior pituitary gland. Both Snell and Ames mice exhibit a deficiency in release of pituitary hormones (growth hormone, thyroid stimulating hormone and prolactin), greatly reduced body size, reduced fertility, increased adiposity, undetectable circulating IGF-1, and low insulin and glucose levels. The other group of somatotropic axis mutants consists of GH-resistant mice, specifically GH receptor and GH-binding protein (GHR/BP) knockout mouse (Laron mouse) (Zhou et al. 1997) as well as GH-deficient mice, due to a spontaneous mutation (*little* mice) or targeted disruption of the *Ghrhr* gene (GHRH-KO), which encodes the GH-releasing hormone receptor (Alba and Salvatori 2004; Godfrey et al. 1993). Hypopituitary dwarf mice display a 40–70% increase in mean lifespan depending on the specific mutation and sex (Brown-Borg et al. 1996; Flurkey et al. 2001). Even though these mutant mice are deficient in other hormones in addition to GH, their lifespan extension seems to be primarily due to GH deficiency, as the lifespan of Ames dwarf mice treated with GH reverts back to that of control animals (Panici et al. 2010).

Hypopituitary, GH deficient and GH resistant mice have been extensively characterised with respect to their metabolic phenotypes, over the years (Bartke and Westbrook 2012). Ames mice, as wells as GHR-KO mice, display increased adiposity, hypoinsulinemia and hypoglycaemia, increased adiponectin levels and reduced serum lipids. Interestingly, visceral fat from GHR-KO mice has been shown to produce higher levels of adiponectin, which likely explains the improved insulin sensitivity and glucose tolerance in the face of increased adiposity (List et al. 2011). Indeed removal of visceral fat from GHR-KO mice resulted in reduced insulin sensitivity in contrast to wild type mice, in which the same intervention improved insulin sensitivity (Masternak et al. 2012). Furthermore, indirect calorimetry experiments revealed increased oxygen consumption and reduced respiratory quotient indicating that these mutants have elevated oxidative metabolism and preferentially utilise lipids as fuel (Westbrook et al. 2009). Such properties can explain the beneficial metabolic parameters, i.e. improved insulin sensitivity and glucose homeostasis of these dwarf mutants. Interestingly, increased respiration was evident only under standard vivarium temperature (commonly 23 °C) whereas under mouse thermoneutral conditions (30 °C) there was no difference (Bartke and Westbrook 2012). This suggests that the small body size of these mutants could be the key determinant of their metabolic phenotype, as it necessitates an increased metabolic rate in order to maintain body temperature.

The extent to which reduction in the IGF-1 accounts for the life extending effects of GH-deficient and GH-resistant mice is not fully clear, but both GH-deficient and GH-resistant mice consistently show more robust lifespan extension than IGF-1-deficient mice (Bartke 2009). Gene inactivation of the IGF-1 receptor in homozygosity is perinatally lethal, but haploinsufficiency of the IGF-1 receptor (IGF-1R^+/−^) resulted in enhanced stress resistance and lifespan extension by 33% in female, but not male, mice (Holzenberger et al. 2003). Another study conducted independently, found a lifespan extension of only 4.7% in female IGF-1R^+/−^ mice, though it confirmed the previously reported stress resistance (Bokov et al. 2011). In terms of metabolic phenotypes, the latter study demonstrated that over ageing the mice developed insulin resistance and the males glucose intolerance as well. A follow up study from the Holzenberger lab, demonstrated that the magnitude of the lifespan extension effect, but not stress resistance, was depended on genetic background (Xu et al. 2014). In terms of metabolic phenotypes, the study corroborated the development of insulin resistance in male mice. Thus, downregulation of IGF-1 signalling does not seem to offer the same beneficial effects with GH deficiency/resistance. Therefore, GH deficiency/resistance likely mediates its life extending effects through mechanisms distinct from IGF-1 deficiency to a large extent.

The neuroendocrine axis of growth control has been implicated in human ageing as well. GH levels drop with age, a process known as somatopause. Certain manifestations of tsomatopause, such as reduction of muscle mass, causing age-related sarcopenia, and increase in visceral adiposity are partly reversible by GH treatment (Rudman et al. 1990). Hence, GH replacement therapy has been proposed as an anti-ageing intervention. However, the findings from research in GH deficient/resistant mouse mutants do not support such an effect of GH and on the contrary suggest that such an intervention could have a negative impact on human ageing (Bartke 2008). Also, in humans there is an apparent negative correlation between height and longevity, thus suggesting a potential role of GH and IGF-1 regulated growth in promoting ageing (Samaras and Storms 1992). There is also genetic evidence implicating the activity of the somatotropic axis in human ageing. Allele frequency studies have reported that polymorphic variants of genes related to GH synthesis, IGF-1 signalling and insulin action change in frequency with age. Bonafè et al. found that allele A of IGF-1R is associated with low plasma IGF-1 and is more frequent among long-lived people (Bonafe et al. 2003). Moreover, van Heemst et al. found that women carrying a SNP variant of the GH1 gene for human growth hormone are 2 cm shorter and exhibit a 0.80-fold reduced mortality (van Heemst et al. 2005). Furthermore, female offspring of centenarian Ashkenazi Jews were found to be heterozygous for loss-of-function mutations in the gene encoding IGF-1R (Suh et al. 2008). However, such evidence is essentially correlative and it does not reveal the underlying mechanisms of lifespan extension. Body size per se is not necessarily a determinant of lifespan extension. Reduced susceptibility to pathologies such as cancer could underlie the lifespan extending effect in this context (de Magalhaes and Faragher 2008). This notion is further supported by the recent report that male carriers of a common GHR allele lacking exon 3 (d3-GHR) live longer despite being taller than carriers of the wild-type allele (Ben-Avraham et al.  2017). Interestingly, a study that monitored 99 Ecuadorian GH receptor deficient dwarfs for 22 years, has shown that they were less susceptible to cancer and obesity, but not long-lived (Guevara-Aguirre et al. 2011). Therefore, diminished somatotropic signalling can have beneficial metabolic effects, even in the absence of lifespan effects in humans.

### Insulin/IGF-1 signalling pathway and metabolic ageing

During the 1980s and 1990s, mutagenic screen studies in *C. elegans* identified the first long-lived mutants, where single mutations produced large effects on lifespan. Worms with loss-of-function mutations in genes in the IIS pathway showed remarkable increases in mean and maximum lifespan and maintained a youthful morphology for longer (Kenyon 2011). Therefore, the IIS pathway was the first signalling pathway shown to play a pivotal role in the ageing process. Indeed, loss-of-function mutation of daf-2, the worm ortholog of the insulin/IGF-1 receptor, resulted in large increases in the lifespan of worms. In contrast, ubiquitous knock out of the Insulin Receptor (IR) gene in mice resulted in neonatal lethality (Accili et al. 1996). Moreover, a severely diminished IR gene function in humans causes Dohonue syndrome (leprechaunism) (Kitamura et al. 2003). Hence, it was initially thought that the role of IIS in the regulation of the ageing process could not be evolutionarily conserved and that the activity of the IIS pathway was unlikely to promote ageing in mammals. However, there is now ample evidence that reduction of the IIS pathway can delay ageing and improve the metabolic profile of mammalian species in late life. The first evidence was provided from the study of mice with adipose tissue-specific knockout of the insulin receptor (FIRKO mice, Fat-specific Insulin Receptor KnockOut). FIRKO mice were protected against age-related obesity and exhibited an 18% increase of mean lifespan in both sexes (Bluher et al. 2003). FIRKO mice displayed reduced insulin levels and resistance to age-related glucose intolerance (Bluher et al. 2002). More recently, extension of the maximal lifespan of heterozygous mice for a ubiquitous null mutation of the insulin receptor (IR-KO^+/−^) has been reported for male, but not female, mice (Nelson et al. 2012). Conversely, reduction of circulating insulin levels by targeted disruption of insulin genes has recently been reported to extend lifespan in female, but not male mice (Templeman et al. 2017). This is an example of how sex can differentially influence the effects of genetic interventions even on closely positioned components of signalling pathways.

Ubiquitous knock-out of the Insulin Receptor Substrate-1 (IRS1) has also been shown to extend lifespan (by 32% in females, weaker effect in males) and improve a wide range of markers of ageing such as immune and motor system dysfunction and bone and skin deterioration (Selman et al. 2008, 2011). IRS1 KO mice, although insulin resistant, display improved glucose tolerance at old age. Although Selman et al. found no lifespan effects in ubiquitous IRS2 heterozygous knockout mice (IRS2^+/−^), another study reported that both ubiquitous IRS2^+/−^ and brain-specific IRS2 KO mice exhibited lifespan extension (Taguchi et al. 2007). Interestingly, ubiquitous IRS2^+/−^ mice were found to be more insulin sensitive than wild-type littermates, but brain-specific IRS2 KO were insulin resistant and glucose intolerant.

A key effector molecule in IR is PI3K. The principal IIS-responsive mammalian isoform of PI3K is p110α (Foukas et al. 2006). Hence, mice heterozygous for a kinase-dead knock-in mutation in the gene encoding p110α (p110α KI) displayed insulin resistance and moderate glucose intolerance at young age. However, this partial inactivation of PI3K p110α exerted a protective effect in the long-term so that aged p110α KI mice were leaner and manifested an improved metabolic profile compared to their wild-type littermates (Foukas et al. 2013). These effects were more prominent in male mice. Consistent with this, male p110α KI mice showed a modest (approx. 7%) extension in their median lifespan. These findings are in line with the phenotypes reported for mice with systemic overexpression of the tumour suppressor phosphatase and tensin homolog (PTEN), which counteracts the activity of PI3K. PTEN transgenic (PTEN-Tg) mice exhibited increased energy expenditure, decreased adiposity, improved insulin sensitivity upon high-fat feeding or with aging, and extended lifespan (Ortega-Molina et al. 2012). High levels of expression of the uncoupling protein 1 (UCP1) in the brown adipose tissue (BAT) of PTEN transgenic mice resulted in enhanced nutrient burning capacity and reduced adiposity and associated pathologies. All the above evidence supports the notion that downregulation of the insulin signalling pathway can have important beneficial metabolic effects in mammals.

### mechanistic Target of Rapamycin (mTOR) pathway and metabolism

The mTOR signalling pathway is evolutionarily conserved and integrates nutrient availability, energy status and growth factor signalling in the control of cell growth and proliferation. It regulates a multitude of cellular processes such as mRNA translation, metabolism, autophagy and stress resistance (Cornu et al. 2013; Kapahi et al. 2010; Laplante and Sabatini 2012). The mTOR protein kinase is distributed in two distinct complexes, mTOR Complex 1 and 2 (mTORC1 and 2), each with distinct functions and substrates. mTORC1 consists of mTOR, mammalian lethal with sec-13 protein 8 (mLST8, also known as GβL), and regulatory-associated protein of TOR (Raptor). Additional components include DEP-domain-containing mTOR-interacting protein (DEPTOR) and proline-rich Akt substrate 40 kDa (PRAS40). mTORC1 is acutely sensitive to rapamycin and regulates ribosomal protein biogenesis, protein translation and autophagy. mTORC2 is composed of mTOR, rapamycin-insensitive companion of mTOR (Rictor), a G protein beta subunit-like associated to mTOR (mLST8), and stress-activated protein kinase-interacting protein 1 (mSIN1) (Cornu et al. 2013; Laplante and Sabatini 2012). S6K1 and 4E-BP1 are two well-characterised substrates of mTORC1 (Ma and Blenis 2009), whereas the hydrophobic motif phosphorylation site (S473) of Akt is a key substrate for mTORC2 (Sarbassov et al. 2005). Both mTORC1 and mTORC2 are activated by growth factors. mTORC1 activity is also modulated by availability of aminoacids and by energy levels through input from AMPK. Hence, mTORC1 is a central signalling node that integrates multiple inputs to regulate biological responses such as protein and lipid synthesis, autophagy and cell cycle progression. mTORC2 has been implicated in cytoskeletal organisation, cell survival and metabolism; in the latter to a large extent via its Akt phosphorylating activity.

Genetic or pharmacological inhibition of mTOR signalling has been found to extend the lifespan of invertebrate species including yeast, nematodes, fruit flies and mice (Kapahi et al. 2010; Lamming et al. 2013). Indeed, deletion of S6K1 protects against diet-induced obesity, enhances insulin sensitivity and increases lifespan in mice (Selman et al. 2009; Um et al. 2004). Moreover mice expressing a hypomorphic allele of mTOR and mice heterozygous for both mTOR and mLST8, are also long-lived (Lamming et al. 2012; Wu et al. 2013). Furthermore, it has recently been shown that long-lived heterozygous Akt1 mutants exhibit decreased mTORC1 activity (Nojima et al. 2013). A role for mTORC2 in the regulation of lifespan is still largely uncertain, but it could potentially have an influence through its role as a modulator of mTORC1 signalling.

The mTOR and IIS pathways are closely linked in the regulation of energy metabolism and glucose homeostasis (Zoncu et al. 2011). Early genetic approaches attempting ubiquitous inactivation of mTOR in mice resulted in early embryonic lethality and therefore did not contribute to the study of mTOR in metabolic regulation (Gangloff et al. 2004; Murakami et al. 2004). Hence, tissue-specific mutagenesis has been applied in order to study the roles of the two mTOR complexes in metabolic tissues, which often resulted in disparate metabolic effects depending on the targeted tissue (Table 1). Tissue-specific disruption of mTOR signalling pathway components has been shown to induce differential effects on the metabolic profile in rodents, summarised in (Polak and Hall 2009). Deletion of Raptor, a component of mTOR C1, specifically in the adipose tissue protected against diet-induced obesity (Polak et al. 2008), whereas deletion of Raptor in skeletal muscle was deleterious, causing severe muscular dystrophy (Bentzinger et al. 2008). Overexpression of a dominant-negative version of Raptor in the liver improved insulin sensitivity (Koketsu et al. 2008), whereas inhibition of mTOR by rapamycin reduced the production and release of insulin by pancreatic islets, leading to hypoinsulinemia and glucose intolerance (Zahr et al. 2008). Also, aminoacid-induced activation of mTOR or activation of S6K in the hypothalamus decreased food intake and body weight (Blouet et al. 2008; Cota et al. 2006). Similar approaches targeting mTORC2 in the adipose tissue by disruption of the Rictor gene resulted in larger body mass due to organ hypertrophy and hyperinsulinemia, but normal glucose tolerance, under standard chow feeding (Cybulski et al. 2009). These findings have been largely corroborated by another study utilising a similar targeting approach, with the exception of the glucose tolerance, which in the later study was found to be impaired (Kumar et al. 2010). From the above data, it becomes evident that the effect of downregulation of mTOR signalling on metabolism is difficult to predict. Pharmacological inhibition of mTOR with rapamycin apparently results in negative metabolic effects though these largely depend on the dosing regimen (see below). And hypomorphic mTOR expression has no metabolic phenotypes despite lifespan extension (Wu et al. 2013). Therefore, in the case of the mTOR pathway, a relation between body metabolic homeostasis and longevity is not obvious.

**Table 1:** Metabolic phenotypes of mTORC1/2 conditionally mutated mice

Component	Tissue	Metabolic phenotypes	References
mTOR	Systemic hypomorphic (mTOR^Δ/Δ^ mice)	Normal insulin sensitivity and glucose tolerance, and energy expenditure	Wu et al. (2013)
mTOR	Skeletal muscle	Severe myopathy, increased muscle glucose uptake and glycogen accumulation, but normal glucose and insulin tolerance	Risson et al. (2009)
Raptor	Adipose tissue	Leanness in the face of reduced physical activity and unaffected caloric intake, protection against diet-induced obesity, improved insulin sensitivity, elevated energy expenditure due to mitochondrial uncoupling	Polak et al. (2008)
Raptor	Liver	Normal glucose tolerance	Lamming et al. (2012)
Rictor	Liver, liver-specific Rictor knockout (LiRiKO) mice	Constitutive gluconeogenesis, impaired glycolysis and lipogenesis, systemic hyperglycemia, hyperinsulinemia, and hypolipidemia	Hagiwara et al. (2012)
Rictor	Adipose tissue	Mice hyperinsulinemic, but glucose tolerant	Cybulski et al. (2009)
Rictor	Adipose tissue	Glucose intolerance, marked hyperinsulinemia, insulin resistance in skeletal muscle and liver and hepatic steatosis	Kumar et al. (2010)
Rictor	Skeletal muscle	Glucose intolerance, but increased basal glycogen synthase activity in muscle	Kumar et al. (2008)

Mutants for which systemic metabolic phenotypes have been reported

### Insulin sensitivity and longevity

Ageing is associated with a reduction in insulin sensitivity both in humans and rodents (Basu et al. 2003; Escriva et al. 2007). Age-related insulin resistance is thought to be a result of increased visceral adiposity with age progression. Consistent with this notion, removal of visceral adipose tissue improves insulin sensitivity in mice (Gabriely et al. 2002). Adipose tissue development and function is highly dependent on sex steroids (White and Tchoukalova 2014) and therefore it is thought that differences in adipose tissue distribution between sexes would produce differences in development of insulin resistance, however there is no clear consensus about this (Kim and Reaven 2013).

The potential effect of insulin sensitivity on lifespan is still unclear (Barzilai and Ferrucci 2012). In humans, insulin resistance is accompanied by compensatory hyperinsulinemia and has clearly been implicated as a risk factor for multiple age-related diseases. Consistent with this, calorically restricted rodents and a number of long-lived mice (e.g. GH deficient/resistant mutants, FIRKO mice, S6K KO mice) display enhanced insulin sensitivity. However, as mentioned above, attenuation of the insulin signalling cascade via genetic inactivation of key IIS pathway intermediates has been associated with lifespan extension in model organisms (Kenyon 2010). Moreover, lifespan extension has been reported in mouse mutants for the IIS or mTOR pathways with normal (Lamming et al. 2012; Nojima et al. 2013; Wu et al. 2013) or reduced (Selman et al. 2008; Taguchi et al. 2007) insulin sensitivity. This implies that enhanced insulin sensitivity is not a requisite for extended longevity. However, insulin resistant mutants, such as IRS1^−/−^ and PI3K p110α KI mice, display improved glucose tolerance compared to wild-type littermates at old age (Foukas et al. 2013; Selman et al. 2008). As mentioned in the Introduction, hyperglycemia contributes to a large extent to tissue damage and old age frailty. Therefore, improved glucose tolerance at old age might be a better indicator of a metabolic effect, which might translate to enhanced longevity, than insulin sensitivity.

### Sirtuins and metabolic effects of sirtuin activation

Sirtuins are a family of nicotinamide adenine dinucleotide (NAD^+^)-dependent histone deacetylases (Chang and Guarente 2014; Houtkooper et al. 2012). There are seven mammalian sirtuins (SIRT1-7) that differ in their tissue distribution, subcellular localisation, enzymatic activity and substrate specificity. In addition to histones, they also deacetylate transcription factors and other cellular proteins affecting gene expression activity. Their implication in lifespan regulation emerged when increasing the dosage of the sirtuin Sir2 was found to extend replicative lifespan in yeast (Kaeberlein et al. 1999). Various lines of evidence support the notion that sirtuins mediate the effects of calorie restriction to a large extent (Guarente 2011). They have also been shown to extend the lifespan of worms, flies and mice. The lifespan extending effects of sirtuin activation have been disputed, but the improvements in healthspan seem to be robust (Houtkooper et al. 2012). Sirtuins mediate various beneficial effects on metabolic tissues, such as reduced glycolysis and increased fatty acid oxidation in liver and muscle, reduced hepatic lipogenesis, adipose tissue browning and fat mobilisation (Chang and Guarente 2014). SIRT1, the principal mammalian sirtuin exerting metabolic effects, has been shown to be activated via the AMPK energy sensing pathway in skeletal muscle (Canto et al. 2010). Therefore, the sirtuin family provides promising therapeutic targets in metabolic diseases, such as age-related obesity and diabetes, and a number of sirtuin activators have been identified or developed. The natural polyphenol compound resveratrol has received great attention as a sirtuin activator with significant beneficial metabolic effects in mice fed a high-fat diet and in obese humans (Baur et al. 2006; Lagouge et al. 2006; Timmers et al. 2011). Resveratrol’s mechanism of action as a sirtuin activator has been reported to be indirect, through activation of energy sensing pathways and modulation of cAMP and NAD^+^ levels, according to some studies (Park et al. 2012; Um et al. 2010). However, later studies demonstrated that the substrate specificity of SIRT1 is sequence specific thus explaining the lack of activation by resveratrol against a number of substrates (Hubbard et al. 2013; Lakshminarasimhan et al. 2013). A number of synthetic SIRT1 activators have been developed and tested in preclinical trials (Carafa et al. 2016; Hubbard and Sinclair 2014; Sinclair and Guarente 2014). Moreover, some positive results have been reported from early stage clinical trials in inflammatory and metabolic disorders (Bonkowski and Sinclair 2016). However, a more detailed evaluation of their potential for clinical application remains to be seen.

## Pharmacological interventions in healthspan extension

The demonstration that administration of the mTOR inhibitor rapamycin late in life can extend the lifespan of mice was a ground-breaking development in the biology of ageing, as it provided solid proof-of-principle that pharmacological treatment of ageing is possible (Harrison et al. 2009). It has boosted efforts to produce pharmacological agents to improve healthspan by combating age-related disease. This section summarises evidence supporting a potential effect of pharmacological agents, known to modulate the activity of signalling pathways implicated in regulation of healthspan and lifespan, on metabolic homeostasis. Specifically, the mTOR pathway inhibitor rapamycin, PI3K inhibitors (IIS pathway), somatoropic axis inhibitors and metformin (indirect activator of AMPK) are discussed in more detail. Sirtuin activators were briefly discussed above and in more detail in the literature cited therein.

### Rapamycin

Rapamycin acts as an allosteric inhibitor of mTORC1 by forming a gain-of-function complex with the 12 kDa FK506-binding protein (FKBP12). Rapamycin has immunosuppressive and anti-proliferative properties in mammalian cells. It was approved as an immuno-suppressant in 1999. In recent years, interest has focused on the potential of rapamycin and its analogues (rapalogues) as anticancer drugs (Wander et al. 2011). Rapamycin has been reported to have both positive and negative effects on mammal metabolism. In obesity, the mTOR pathway is hyper-activated in the adipose tissue thus leading to increased lipogenesis, reduced lipolysis and fat accumulation. To limit its over-activation, mTORC1 blocks insulin signalling through a S6K-mediated feedback loop destabilising IRS (Harrington et al. 2004; Shah et al. 2004) and through direct phosphorylation and stabilisation of Grb10 (Hsu et al. 2011), provoking an insulin resistance state. Acute mTOR inhibition through rapamycin treatment improves insulin sensitivity in vitro and in vivo by disrupting this negative feedback loop of insulin signalling (Krebs et al. 2007; Tremblay and Marette 2001). Also rapamycin protects from diet-induced obesity and prevents weight gain in mice (Chang et al. 2009a; Makki et al. 2014). In rats and humans, it reduces age-related body weight gain when administered 3 times per week (Rovira et al. 2008). However, glucose intolerance and insulin resistance have been observed in a few strains of rodents treated daily with high doses of rapamycin. In fact, rapamycin increases lipolysis in the adipose tissue in rats, causing pronounced hyperlipidemia (Houde et al. 2010). Also, a 2-week rapamycin treatment aggravates hyperglycemia in a diabetic mouse model (Fraenkel et al. 2008) and similarly, rapamycin administration (6 weeks) exacerbates glucose intolerance in diet-induced obese KK/HIJ mice (Chang et al. 2009b). These controversial findings on the insurgence of insulin resistance might be explained by the duration and the doses of rapamycin treatment (Fang et al. 2013). Rapamycin could therefore still be a viable pharmacological option to promote beneficial metabolic effects in humans pending definition of appropriate dosing regimens.

### GH/IGF1 axis inhibitors

Research in a number of GH deficient/resistant mice as well as in an Ecuadorian human GH receptor-deficient population, has clearly demonstrated that downregulation of this pathway exerts beneficial metabolic effects. Compounds targeting the activity of the somatotropic axis have been used in the treatment of acromegaly. Somatostatin analogues have been used to supress GH secretion, but with limited efficacy and substantial side-effects (Parkinson et al. 2002). Somatostatin inhibits secretion of insulin and it is therefore unlikely to have beneficial metabolic and healthspan effects. However, pegvisomat, a GH receptor antagonist, has demonstrated efficacy and an acceptable safety profile (van der Lely et al. 2012). Beneficial metabolic effects of short- to medium- term administration of pegvisomat in type-1 diabetes and acromegaly patients, respectively, have been reported (Lindberg-Larsen et al. 2007; Thankamony et al. 2014). Pegvisomat, is therefore a compound that could potentially be tested for healthspan effects upon long-term administration in humans, although logistic constraints related to high costs, make it an unlikely candidate as a widely available therapeutic modality.

### PI3K inhibitors

As discussed above, long-lived IIS mutant mice, such as IRS1 KO (Selman et al. 2008), PTEN-Tg mice (Ortega-Molina et al. 2012) and PI3K p110α KI mice (Foukas et al. 2013) display a metabolic improvement at old age. Therefore it is reasonable to hypothesise that use of inhibitors against ‘druggable’ components of this pathway could represent a strategy in prevention or treatment of metabolic diseases associated with old age (such as obesity and type-2 diabetes) and in healthspan extension. A number of inhibitors against PI3Ks are currently available, mainly through efforts to target these enzymes in oncology. Proof of principle that PI3K inhibitors can also be useful in metabolic disease has been provided by a recent study (Ortega-Molina et al. 2015). Long-term administration of low doses of two pharmacological inhibitors of PI3K, CNIO-PI3Ki and GDC-0941, has been reported to reduce the adiposity and body weight of obese mice and rhesus monkeys. The same treatment did not affect adiposity of mice fed a standard chow. CNIO-PI3Ki is a small molecule ATP competitive dual inhibitor of the PI3K isoforms p110α and p110δ. Further testing for isoform specificity, using p110α and p110δ discriminating compounds, revealed that the anti-obesity effect was particularly prominent upon inhibition of p110α, the principal insulin activated isoform, although inhibition of p110δ did contribute to the overall effect (Lopez-Guadamillas et al. 2016). p110δ is highly expressed in leukocytes and its involvement was possibly due to an anti-inflammatory effect of p110δ inhibition, as inflammation is a well-established contributing factor in metabolic pathology. These findings suggest that pharmacological inhibition of PI3K could be an effective and safe anti-obesity intervention to prevent or reverse metabolic syndrome in humans.

### Metformin

Metformin is a biguanide that has extensively been used in the treatment of type 2 diabetes. It works by decreasing glucose production in the liver, augmenting glucose utilisation by body tissues and increasing sensitivity to insulin. Metformin has been used for more than 60 years, it is safe and has also been reported to slow aging in *C. elegans* (Pryor and Cabreiro 2015). Metformin-treated worms not only live longer, but also stay healthier for longer (Onken and Driscoll 2010). Metformin administration increases the lifespan of mice by nearly 6% and improves various markers of healthspan (Martin-Montalvo et al. 2013). Also diabetic patients treated with metformin live longer than non-treated non-diabetic control subjects (Bannister et al. 2014). Therefore, metformin might have substantial beneficial effects on human healthspan. To test this idea, metformin is set to enter a ground breaking human trial as a potential anti-aging drug (Barzilai et al. 2016). A clinical trial called Targeting Aging with Metformin, or TAME will involve the administration of metformin to 3,000 people aged 70–80 years (at roughly 15 centres around the United States), who already have one or two of three conditions (cancer, heart disease or dementia) or are at risk of them. The participants will be monitored to test whether the medication prevents or delays development of diseases they do not already have, as well as of diabetes.

## Conclusion and perspective

Recent advances in the elucidation of signalling pathways that modulate the rate of ageing have made it possible to study the effects of their manipulation on various pathologies associated with advanced age in model organisms. However, a number of confounding factors complicate the predictions for translational potential of the findings from model organism to humans (de Magalhaes 2014). Most of the research in the genetics of ageing and in testing interventions has used inbred animals with identical genetic backgrounds. Such models do not reflect the situation of human populations that are genetically heterogeneous. In many cases, the responses under investigation have been extremely variable between different strains. A prominent example is calory restriction in mice, where the outcome can vary from life extension to life shortening depending on the genetic background (Liao et al. 2010). Furthermore, as alluded at various places above, in many cases, genetic interventions have stronger or even restricted effects on one sex. It is conceivable that, as is the case in other diseases, notably in cancer, in age-related diseases there will not be a cure suitable for everyone, but treatments should be personalised based on individual genetics. Pharmacologic interventions might have to match the genetic and epigenetic make-up as well as the sex of individuals. To this end, continuous advances in next generation sequencing and human genetics, together with development of advanced bioinformatic methods, similar to those used to correlate longevity genes to age-related disease genes and to drugs (Fernandes et al. 2016), could make it possible to predict the likelihood of specific interventions to be effective in combating age-related diseases in particular individuals.

There is now substantial evidence that pharmacological interventions in the IIS, mTOR and AMPK pathways can have beneficial metabolic effects in mammalian organisms. As discussed above, interventions within these signalling pathways affect metabolic homeostasis, which appears to be a key determinant of healthspan. A number of compounds targeting these pathways have demonstrated good tolerability and substantial beneficial metabolic effects through preclinical testing in mice (Table 2). Whether such compounds will only have beneficial metabolic effects or more generalised healthspan effects will be of great interest to determine, but it will certainly be more challenging and it will require development of suitable biomarkers. Nevertheless, the application of signalling pathway inhibitors for prevention and treatment of age-related obesity and insulin resistance seems within grasp based on very promising results from preclinical stage testing. Exciting trials are under way to assess the effects of such compounds, such as the rapamycin trial in pet dogs (Kaeberlein et al. 2016) and the metformin trial in humans (Barzilai et al. 2016). Ongoing research in independent academic laboratories along with larger scale programmes such as the National Institute of Aging Intervention Testing Programme (NIA ITP) (https://www.nia.nih.gov/research/dab/interventions-testing-program-itp) have made great contributions to this effort and will likely identify additional compounds with potency to improve healthspan and lifespan to be subsequently tested in human trials. These trials hold great promise and a positive outcome would be a great return for the extensive efforts invested in research in the field of biology of ageing. Such developments are eagerly awaited by the respective scientific community and they are certain to be welcome by the general public.

**Table 2:** Compounds with demonstrable effectiveness against metabolic conditions in preclinical testing

Compound	Target	Effects	References
CNIO-PI3Ki	PI3K	Anti-obesity	Ortega-Molina et al. (2015)
Metformin	AMPK and other targets	Improved insulin sensitivity and plasma lipid profiles	Martin-Montalvo et al. (2013)
Rapamycin	mTOR	Reduced body weight, enhanced insulin sensitivity	Fang et al. (2013)
SIRT1720	Allosteric SIRT1 activator	Lower body weight Improved glucose tolerance and insulin sensitivity	Mitchell et al. (2014)

List of compounds with activity on metabolic signalling pathways shown to be well tolerated and to induce beneficial metabolic effects upon chronic administration in mammalian models
